# In Vitro Fermentation of *Pleurotus eryngii* Mushrooms by Human Fecal Microbiota: Metataxonomic Analysis and Metabolomic Profiling of Fermentation Products

**DOI:** 10.3390/jof9010128

**Published:** 2023-01-16

**Authors:** Paris Christodoulou, Marigoula Vlassopoulou, Maria Zervou, Evangelos Xanthakos, Panagiotis Moulos, Georgios Koutrotsios, Georgios I. Zervakis, Evangelia N. Kerezoudi, Evdokia K. Mitsou, Georgia Saxami, Adamantini Kyriacou, Vasiliki Pletsa, Panagiotis Georgiadis

**Affiliations:** 1 National Hellenic Research Foundation, Institute of Chemical Biology, 11635 Athens, Greece; 2 Department of Biochemistry and Biotechnology, University of Thessaly, 41500 Larissa, Greece; 3 Department of Nutrition and Dietetics, Harokopio University, 17676 Kalithea, Greece; 4 Department of Biotechnology, Agricultural University of Athens, 11855 Athens, Greece; 5 Biomedical Sciences Research Center Alexander Fleming, 16672 Vari, Greece; 6 Laboratory of General and Agricultural Microbiology, Agricultural University of Athens, 11855 Athens, Greece; 7 School of Medical Sciences, Örebro University, SE-701 82 Örebro, Sweden

**Keywords:** *Pleurotus eryngii* mushrooms, in vitro static batch fermentation, gut microbiota, metataxonomics, metabolomics

## Abstract

Edible mushrooms contain biologically active compounds with antioxidant, antimicrobial, immunomodulatory and anticancer properties. The link between their anticancer and immunomodulatory properties with their possible prebiotic activity on gut micro-organisms has been the subject of intense research over the last decade. Lyophilized *Pleurotus eryngii* (PE) mushrooms, selected due to their strong lactogenic effect and anti-genotoxic, immunomodulatory properties, underwent in vitro static batch fermentation for 24 h by fecal microbiota from eight elderly apparently healthy volunteers (>65 years old). The fermentation-induced changes in fecal microbiota communities were examined using Next Generation Sequencing of the hypervariable regions of the 16S rRNA gene. Primary processing and analysis were conducted using the Ion Reporter Suite. Changes in the global metabolic profile were assessed by ^1^H NMR spectroscopy, and metabolites were assigned by 2D NMR spectroscopy and the MetaboMiner platform. PLS-DA analysis of both metataxonomic and metabolomic data showed a significant cluster separation of PE fermented samples relative to controls. DEseq2 analysis showed that the abundance of families such as *Lactobacillaceae* and *Bifidobacteriaceae* were increased in PE samples. Accordingly, in metabolomics, more than twenty metabolites including SCFAs, essential amino acids, and neurotransmitters discriminate PE samples from the respective controls, further validating the metataxonomic findings.

## 1. Introduction

Edible mushrooms have been used for centuries in traditional medicine as enhancers of wellbeing [1]. Current research has identified many of their health-promoting properties, ranging from antioxidant [2], antimicrobial [3], genoprotective [4] and anticancer [5] activities to immune enhancement [6,7] and prebiotic action [8]. These beneficial effects have been attributed to a plethora of biomolecules that are found in mushrooms, especially polysaccharides, due to their possible prebiotic activity on gut microorganisms [9].

The human body is inhabited internally and externally by vast numbers of microbes, predominantly residing on the skin, oral cavity, conjunctiva, vagina, lungs and across the gastrointestinal tract [10]. The diverse communities of bacteria, archaea, eukaryotes, and viruses colonizing the latter are collectively referred to as gut microbiota (GM) and comprise the largest microorganism population harbored by the human host [11,12]. GM represent a total mass of approximately 0.2 kg [10], while metagenomic analyses have revealed that more than 99% of the genes encoded by GM are bacterial, while the remaining 1% corresponds to genes belonging to archaea [13]. These data reflect the prevalence of bacteria over archaea and eukaryotes in terms of GM cell numbers, with recent estimates suggesting that about 3.8 × 10^13^ bacterial cells live in the average person’s gut [10], which translates to a 1:1 ratio compared to total human cells and tenfold the number of the nucleated ones (approximately 0.3 × 10^13^) [10].

The GM comprise a dynamic and personalized microbial assembly affected by multiple factors, including diet, age, sex, genetics, consumption of antibiotics and other drugs, geographic location, environmental toxicants and overall lifestyle [14,15]. Its composition is remarkably diverse and demonstrates great variability across individuals, as more than 1800 genera and 15,000–36,000 species are potential gut inhabitants [16], with the distal gut being dominated by the phyla *Bacteroidetes* and *Firmicutes* (>90%) [13,17]. Notably, there are particular genera and species acting as either beneficial or harmful influencers of the host’s health.

Another important aspect of GM activity is the production of bioactive metabolites through the fermentation of non-digestible nutrients, such as complex carbohydrates [18], the fermentation of which produces short-chain fatty acids (SCFAs), that can be employed as energy sources by the host and also, function as signaling molecules [13,17]. Other GM-derived metabolites are branched-chain fatty acids (BCFAs), amino acids and bile acids [19]. Moreover, recent research has revealed that gut bacteria can biosynthesize de novo essential amino acids from non-specific nitrogen sources and are involved in the digestion of proteins or peptides that are not absorbed in the upper part of the digestive system [20,21,22].

Therefore, taking into account the critical role of GM in numerous functions regulating the host’s wellbeing, ranging from metabolism and homeostasis [23] to preservation of the intestinal epithelium’s integrity [16], immunomodulation [24] and protection from invading pathogens [25], the maintenance of a balanced GM composition where beneficial bacteria prevail in order to avoid dysbiosis and prevent various pathological conditions, is of paramount importance [17,18].

Diet could be employed as a tool to alter GM composition since nutrient fermentation and its products can induce an increase in beneficial bacteria [26]. Indeed, there is evidence indicating that the adoption of new dietary habits for even a short period of 14 days can cause observable changes in an individual’s GM [27]. The identification of dietary factors with prebiotic activity affecting the GM composition and host’s health positively [14], has been the subject of intense research over the last decade with edible mushrooms being a cornerstone of this effort since their genoprotective, anticancer and immunomodulatory properties are possibly linked to their prebiotic-like impact on gut microorganisms [28]. *Pleurotus eryngii*, also known as the “king oyster mushroom”, is a widely cultivated edible mushroom in Europe, North America and Asia. Its fruit bodies are rich in proteins, carbohydrates, unsaturated fatty acids, vitamins, and other nutrients and low in fat, thereby constituting a high-quality, low-calorie food [29]. The polysaccharides of *Pleurotus eryngii* demonstrate antioxidant, anti-tumor, antibacterial, anti-hyperlipidemic, and immunoregulatory activities [30]. So far, a few studies have focused on the impact of the *P. eryngii* carbohydrate fraction on the gut microbiota composition [31,32] while the impact of the whole mushroom, also containing proteins and other bioactive substances on gut microbiota composition and its metabolome has not been investigated thoroughly. To our knowledge, such an approach has been recently applied [33], however, only the SCFA fraction of the metabolome was quantified. Hence, an effort to investigate the beneficial effects of the whole mushroom particularly on the elderly was undertaken. Lyophilized *Pleurotus eryngii* (PE) mushrooms (Basidiomycota, Agaricales), underwent in vitro static batch fermentation for 24 h by fecal microbiota from eight elderly apparently healthy volunteers (>65 years old) and, following the validation of their potent lactogenic [34], anti-genotoxic [4] and immunomodulatory [35] activities, they were further investigated as to their potential prebiotic properties in the context of this study. The fermentation-induced changes in fecal microbiota communities were examined using Next Generation Sequencing of the hypervariable regions of the 16S rRNA gene while, in parallel, the metabolites resulting from this process were identified through ^1^H NMR spectroscopy.

## 2. Materials and Methods

### 2.1. In Vitro Static Batch Culture Fermentations

*Pleurotus eryngii* strain LGAM 216 was isolated from a fruit body (basidiome) collected in Greece, and maintained in the fungal Culture Collection of the Laboratory of General and Agricultural Microbiology (Agricultural University of Athens); strain cultivation and mushroom production conditions were as previously described [4,35]. The lyophilized *P. eryngii* (PE) mushroom powder underwent in vitro static batch fermentation by fecal microbiota from eight apparently healthy volunteers (>65 years old). Fermentations without any additional carbon source were also carried out (negative controls, NC). Fecal donors were apparently healthy subjects (>65 yrs) who met the following inclusion criteria: (a) body mass index (BMI) < 30 kg m^−2^, with no recent weight loss and extreme dietary behaviors; (b) no history of gastrointestinal disease, chronic constipation, chronic/acute diarrhea, autoimmune disease, coronary disease, liver and/or kidney malfunction; (c) no consumption of antibiotics two months before the study; and (d) no consumption of probiotics and/or prebiotics and/or dietary fiber supplements two weeks before the study [34].

Subjects completed a series of questionnaires in relation to sociodemographic parameters (including age, sex, marital status and education level), smoking habits and medical history. Fecal sample collection and in vitro static batch culture fermentation were performed for 8 and 24 h as previously described [4,34,35], however, this current analysis was applied to 0 and 24 h samples only, because the production of SCFAs was enhanced at 24 compared to 8 h [34]. The study groups consisted of 32 samples collected from in vitro cultures. Sample categorization was based on (a) the treatment, namely lyophilized *P. eryngii* mushroom substrate (PE) or no mushroom substrate (NC), and (b) the fermentation impact (0 h—before fermentation/24 h—after fermentation).

### 2.2. 16S Next Generation Sequencing–Metataxonomics

#### 2.2.1. DNA Extraction and Amplification

Samples were collected from the in vitro static batch cultures at 0 h (before fermentation) and 24 h (after fermentation) and genomic DNA (gDNA) was isolated with the QIAamp^®^ DNA Mini Kit (250) (QIAGEN GmbH, Hilden, Germany) as previously described [36,37]. Ten nanograms of gDNA from each sample as measured by nanodrop technology (Hellma traycell; Hellma GmbH & Co. KG., Müllheim, Germany) were used as the template for PCR amplification of hypervariable regions of the 16S rDNA gene with the Ion Torrent 16S Metagenomics kit (Thermo Fisher Scientific, Warrington, UK). To increase the resolving power of 16S rRNA profiling, PCR amplification was performed in two pools, each including specific primer sets. Pool 1 contained primers targeting the V2, V4, V8 regions whereas pool 2 contained those targeting the V3, V6–7 and V9 regions. Indicative amplicon lengths for *Escherichia coli* of the V2, V3, V4, V6–7, V8 and V9 hypervariable regions are 250, 215, 288, 260, 295, and 209, respectively [19]. The PCR was performed under the conditions indicated in the manufacturer’s protocol. After PCR amplification, equal volumes of PCR products from pools 1 and 2 of each sample were combined into a single PCR tube and purified using the Agencourt AmpureXP kit according to the manufacturer’s instructions (BeckmanCoulter, Brea, CA, USA). The concentration of the amplicon mixture was calculated with the use of the Qubit 4 Fluorometer (Invitrogen by Thermo Fisher Scientific, Life Technologies Holdings Pte Ltd., Singapore) and the Qubit 1X dsDNA HS Assay Kit (Invitrogen by Thermo Fisher Scientific, Life Technologies Holdings Pte Ltd., Singapore), following the manufacturer’s instructions.

#### 2.2.2. Preparation of Libraries and Sequencing

One hundred and fifty nanograms of the resulting amplicon mixture were used for library construction with the Ion Plus Fragment Library kit (ThermoFisher Scientific, Life Technologies, Carlsbad, CA, USA) in combination with Ion Xpress™ Barcode Adapters 1–96 Kit (Life Technologies, Carlsbad, CA, USA), following the manufacturer’s instructions. Bead-based cleanup throughout these processes was accomplished with the AMPure^®^ XP Reagent (Beckman Coulter, Inc. Brea, CA, USA). The quantity of the libraries was calculated using a Qubit 4 Fluorometer as mentioned in Section 2.2.1. The quality of the libraries was assessed, and the Qubit-calculated quantity was confirmed by the Agilent TapeStation 4150 (G2992A) with High Sensitivity D1000 ScreenTape^®^ and High Sensitivity D1000 Reagents (Agilent Technologies, Inc., Santa Clara, California, USA). Each library was diluted to a concentration of 26 pM and equal volumes of each library were pooled. The pooled libraries were clonally amplified on nanosized ionosphere particles by emulsion PCR applying the Ion OneTouch™ 2 System (OT2) with the Ion PI™ Hi-Q™ OT2 200 Kit (Life Technologies, Carlsbad, CA, USA), following the manufacturer’s protocol. Sequencing was performed on the Ion Proton sequencer with the utilization of the kits Ion PI Chip Kit V3 (Life Technologies, Carlsbad, CA, USA) and Ion Proton Sequencing 200 Kit (Life Technologies, Carlsbad, CA, USA), with 520 flows following the manufacturer’s instructions.

#### 2.2.3. 16S Sequencing Data Processing and Quality Assessment

Data sequencing, processing and quality assessment was performed based on Karabudak et al. [38] using the automated streamlined software Torrent Suite v5.10.1 (TSS) and Ion Reporter™ Software 5.18.4.0. Torrent Suite was used for sequencing base-calling from the Proton-generated data, and Ion Reporter was used for annotation and taxonomical assignments. The default parameters in the Torrent Suite software include trimming low-quality 3′ ends of reads, filtering out entire reads with pure quality, and removing adapter sequences, polyclonal reads, etc. Ion Torrent Proton-generated sequences were saved as UBAM files and transferred to the Cloud Ion Reporter software.

#### 2.2.4. OTU Clustering

Operational taxonomic unit (OTU) analysis was performed using Ion Reporter Software, which runs Quantitative Insights into Microbial Ecology (QIIME) [39]. Sequences at the single-end mode with a cut-off of 150 bp were mapped. Reads were mapped to two reference databases, the curated MicroSEQ^®^ 16S Reference Library v2013.1 and the curated Greengenes v13.5. The minimum alignment coverage of a read to a sequence in the database in order to assign taxonomy was set at 90%, with an abundance filter set at a minimum of 10 reads. The default cut-off values of 97%, 99% and 0.2% similarity for genus, species and slash IDs reporting percentages were applied respectively.

100,000 to 200,000 reads were finally mapped on average per sample. The mapped reads were assigned to operational taxonomic units (OTUs) and the taxonomic distributions for the consensus data for each sample (according to mapped reads generated from seven hypervariable regions) were constructed by the Ion Reporter to be used for the calculation of relative abundances of microbiota at the taxonomic levels of families and genera. Ion Reporter also provides detailed taxonomic distribution from all hypervariable regions individually, therefore, the datasets from the V3 region, the most commonly used hypervariable region in the literature, were also utilized for comparison purposes.

#### 2.2.5. Alpha Diversity Analysis

Alpha diversity graphics created by QIIME software were exported from the Ion Reporter Software. For alpha diversity analysis, the observed species and the Chao1 and Shannon indexes were generated to analyze species diversity within samples.

### 2.3. NMR-Based Metabolomics

#### 2.3.1. Sample Preparation

Sample preparation was based on an already published protocol, developed in-house [4]. Briefly, 16 samples were freeze-dried under vacuum for 24 h at a constant temperature of 25 °C until dryness. The freeze-dried samples were then stored at −80 °C. The samples were defrosted at ambient temperature 30 min prior to NMR experiments and were then dissolved in 540 μL of phosphate buffer (NaH_2_PO_4_/Na_2_HPO_4_, pH = 7.2) using 60 μL of TSP (0.5 mM) as an internal standard.

#### 2.3.2. NMR Measurements and Data Processing

NMR experiments were performed at 25 °C on a Varian 600 MHz spectrometer using a ^1^H(13C/15N) 5 mm PFG Automatable Triple Resonance probe. The global metabolic profiling of the examined fermented products was assessed, applying 1D NOESY—presat pulse sequence. The NOESY presat experiments were implemented with a mixing time of 200 ms, and solvent suppression by pre-saturating the sample with gammaB1 of 103 Hz for 1 s. Spectra were acquired with 64 k complex data points and 32 scans. Inverse recovery experiments specified the relaxation delay to 5 s. Thirty-seven (37) metabolites were unambiguously assigned with the use of two-dimensional (2D) homo- and hetero-nuclear NMR experiments, tools such as MetaboMiner [40], Chenomx software [Chenomx Inc. Edmonton, Canada], and literature data [41], as previously described [4]. The identified metabolites were further quantified by the application of Chenomx.

#### 2.3.3. Omics Data Statistical Analysis

##### Metataxonomic Analysis

The significance of the differences between groups in the resulting alpha diversity indices was determined using paired samples *t*-test (*p* < 0.05). Supervised partial least-squares discriminant multivariate analysis (PLS-DA) was used for the visual exploration of the samples’ microbiota differential composition (NC vs. PE and pre- and post-fermentation groups). PLS-DA was performed following filtering of the OTU using a threshold of at least 25% of the samples having non-zero values per OTU. For the PLS-DA, data were normalized with mean-centering and division by the standard deviation of each variable. Families and genera with the most significant contribution to composition differences between groups were visualized in the corresponding VIP plots. The quality of the models was described by the goodness-of-fit R2 (0 ≤ R2 ≤ 1) and the predictive ability Q2 (0 ≤ Q2 ≤ 1) values. The PLS-DA results were cross-validated by carrying out permutation tests with 1000 random permutations. For OTU differential abundance testing between the groups, pairwise comparisons were performed with DESeq2 analysis (OmicSoft Studio 11, Qiagen) and the Benjamini–Hochberg false discovery rate (FDR) for multiple comparisons was utilized (*p* < 0.05).

##### Metabolomic Analysis

The derived metabolomics dataset was subjected to statistical analysis using the Metaboanalyst 5.0 platform [42]. Unsupervised (PCA) and Supervised (PLS-DA) multivariate statistical approaches were used for the exploratory data analysis (pre- vs. post-fermentation, NC vs. PE groups). Range scaling (mean-centered and divided by the value range of each variable) was applied to the data. Quality of the models, cross validation and pairwise comparisons were performed as described in Section 2.3.3.

## 3. Results

### 3.1. 16S rRNA Metataxonomic Analysis

Most 16S rRNA metataxonomic analyses reported in the literature are performed by sequencing of the V3 or of the combined V3–V4 regions. However, neither of the regions could identify all detectable genera while the consensus data from all hypervariable regions have been reported to provide more comprehensive results [38]. Therefore, for all the analyses performed, we decided to adopt the consensus microbial composition by combining the OTUs across the seven sequenced hypervariable regions (see Materials and Methods). Nevertheless, the diversity derived from sequencing the V3 hypervariable region alone was also analyzed and compared to the consensus dataset’s results. We confirmed that the diversity of OTU detected using the consensus dataset is greater (Supplementary Figure S1), and DESeq2 analysis showed comparable results for both consensus and V3 datasets (Supplementary Table S1).

Sequencing analysis identified 96 families and 113 genera in all 32 samples. Of them, 41 families and 57 genera were subjected to further analysis, following the exclusion of OTUs detected (non-zero values) in less than 25% of all samples.

Samples were categorized into four groups, NC0, PE0, NC24 and PE24, according to the absence or the presence of *P. eryngii* lyophilized mushroom powder in the fermentation medium (NC and PE, respectively) and the time of sample collection (0 h, pre-fermentation; 24 h, post-fermentation).

DESeq2 analysis for the families and genera between the pre-fermentation NC0 and PE0 groups showed no difference in the microbiota composition between the two conditions (all FDR values were higher than 0.7), as expected, indicating that technical variations due to the handling during the preparation of the NC and PE samples were negligible (Supplementary Figure S2).

#### 3.1.1. Alpha Diversity Analysis

**Figure 1:**
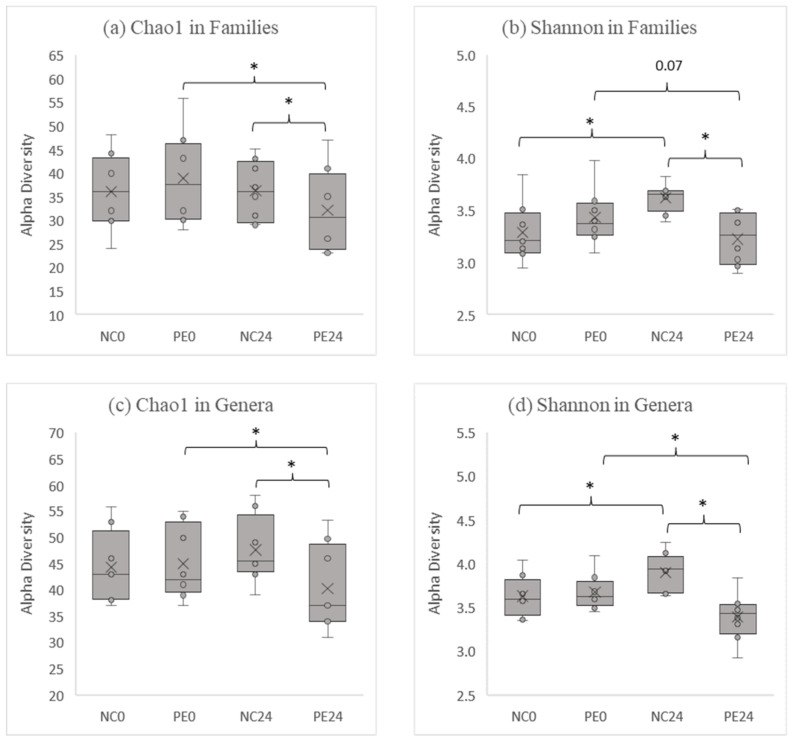
Box plots of Chao1 (**a**,**c**) and Shannon (**b**,**d**) alpha diversity measure estimates at family (**a**,**b**) and genus (**c**,**d**) levels. The X indicates the mean value; the circles indicate the samples’ alpha diversities; ‘*’ indicates a statistically significant difference as measured by paired-samples *t*-test with a *p*-value of < 0.05. NC0: Samples before fermentation in the absence of an additional carbon source (negative controls at 0 h), NC24: Samples after 24 h of fermentation in the absence of an additional carbon source (negative controls at 24 h), PE0: Samples before fermentation in the presence of lyophilized mushroom powder of *P. eryngii*, PE24: Samples after 24 h of fermentation in the presence of lyophilized mushroom powder of *P. eryngii*.

The alpha diversity of the fecal samples before and after fermentation was measured using Chao1 and Shannon indices. The Chao1 index indicates the richness in the diversity of bacterial communities, whereas the Shannon index indicates their evenness along with the richness. Chao1 indices were significantly decreased in the PE post-fermentation (PE24) when compared to the pre-fermentation samples (PE0), in both families (*p* = 0.004; Figure 1a) and genera levels (*p* = 0.023; Figure 1c). Subsequently, both Chao1 indices showed a statistically significant decrease in the PE post-fermentation samples (PE24) in comparison with the respective NC24 post-fermentation ones (Figure 1a,c). Similar findings were also observed when Shannon indices were used (Figure 1b,d). An effect not observed with the Chao1 was that Shannon indices were significantly increased in the control post-fermentation (NC24) when compared to the pre-fermentation samples (NC0), in both family and genus levels.

#### 3.1.2. Microbiota Composition Analysis

**Figure 2:**
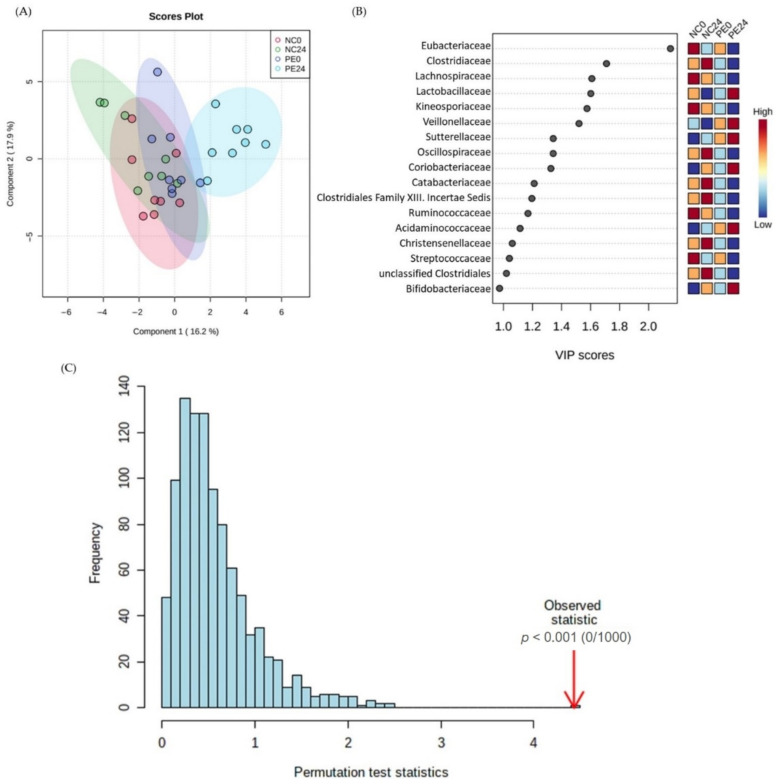
Metataxonomic analysis of families’ abundance in NC and PE in vitro samples both before and after fermentation. (**A**) Partial Least Square Regression-Discriminant Analysis (PLS-DA) scores plot (R2 = 0.69, Q2 = 0.40, accuracy = 0.46), (**B**) VIP scores plot of important families identified by PLS-DA. The colored boxes on the right indicate the relative concentrations of the corresponding family in each group under study, (**C**) PLS-DA model validation by permutation tests based on separation distance. The *p* value based on permutation is *p* < 0.001 NC0: Samples before fermentation in the absence of an additional carbon source (negative controls at 0 h), NC24: Samples after 24 h of fermentation in the absence of an additional carbon source (negative controls at 24 h), PE0: Samples before fermentation in the presence of lyophilized mushroom powder of *P. eryngii*, PE24: Samples after 24 h of fermentation in the presence of lyophilized mushroom powder of *P. eryngii*.

**Figure 3:**
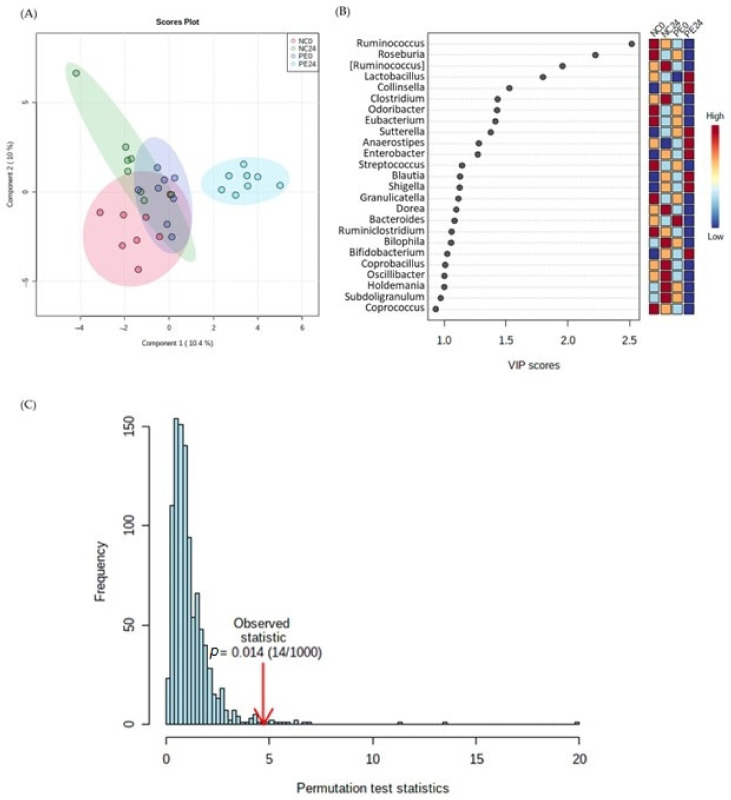
Metataxonomic analysis of genera abundance in NC and PE in vitro samples both before and after fermentation. (**A**) Partial Least Square Regression-Discriminant Analysis (PLS-DA) scores plot (R2 = 0.87, Q2 = 0.42, accuracy = 0.48), (**B**) VIP scores plot of important genera identified by PLS-DA. The colored boxes on the right indicate the relative concentrations of the corresponding family in each group under study, (**C**) PLS-DA model validation by permutation tests based on separation distance. The *p* value based on permutation is *p* < 0.014. NC0: Samples before fermentation in the absence of an additional carbon source (negative controls at 0 h), NC24: Samples after 24 h of fermentation in the absence of an additional carbon source (negative controls at 24 h), PE0: Samples before fermentation in the presence of lyophilized mushroom powder of *P. eryngii*, PE24: Samples after 24 h of fermentation in the presence of lyophilized mushroom powder of *P. eryngii*. The Square brackets ([]) around a genus indicates that the name awaits appropriate action by the research community to be transferred to another genus.

Figure 2 and Figure 3 show the results after the application of supervised (the pre/post-fermentation condition in NC and PE samples was used as the response variable) multivariate analysis (Partial Least Square Discriminant Analysis, PLS-DA) of families’ and genera’s abundance, respectively.

The latter analyses show that the family profile of PE24 samples clustered away from the negative controls before and after fermentation (NC0 and NC24, respectively), as well as its respective pre-fermentation control (PE0) (Figure 2A). The model was validated by permutation test statistics (*p* < 0.001; Figure 2C). The corresponding VIP scores plot (Figure 2B) presents the families with the most significant contribution in the groups’ differentiation, as arises from the model. Indicatively, *Eubacteriaceae*, *Clostridiaceae* and *Lachnospiraceae* were among the families with VIP score >1 that had a lower relative concentration in PE24 in comparison to the rest of the groups (NC0, PE0, NC24), while *Lactobacillaceae* and *Veillonellaceae* were the families that had the highest relative abundance in this group.

A similar clustering pattern was also observed at the genus level of analysis. PLS-DA analysis of genera demonstrated clustering of the PE24 samples along the first component (Figure 3A) with a permutation *p* value at *p* < 0.014 (Figure 3C). Based on the VIP scores plot for genera with VIP score > 1 (Figure 3B), *Lactobacillus* and *Bifidobacterium* were among the genera with the highest relative concentration in PE24. Conversely, *Ruminococcus*, *Roseburia*, *Clostridium*, and *Streptococcus* were among those with the lowest relative concentration in PE24 fecal fermentation samples compared to NC0, NC24 and PE0.

**Figure 4:**
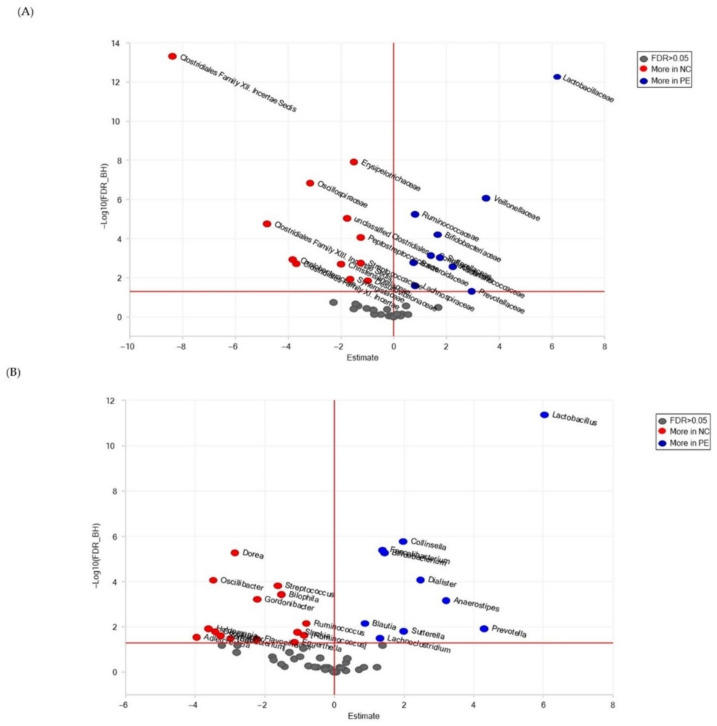
Volcano plots for the comparison of microbiota abundance among fecal samples that underwent 24 h of fermentation with and without the presence of *P. eryngii*. (**A**) families, (**B**) genera. Estimate = log2 fold change; the vertical red line is set to Estimate = 0; the horizontal red line is set to FDR = 0.05.

The effect of the in vitro fermentation of *P. eryngii* on fecal microbiota was also examined by comparing the abundance of the microbiota in the NC24 and PE24 post-fermentation samples at the family and genus levels by utilizing univariate paired DESeq2 analysis. Families and genera with statistically significant differential abundance (FDR < 0.05) are shown in Figure 4 and listed in Supplementary Tables S2 and S3, respectively. The presence of *P.eryngii* in the in vitro fermentation significantly enhanced the abundance of several families, mainly of *Lactobacillaceae*, *Veillonellaceae*, *Prevotellacea*, *Bifidobacteriaceae*, *Coriobacteriaceae*, *Lachnospiraceae*, *Ruminococcaceae* compared to control NC24 samples where *Oxalobacteraceae*, unclassified *Clostridiales*, *Peptostreptococcaceae*, and *Desulfovibrionaceae* were significantly more abundant (Supplementary Table S2). At the genus level, (Figure 4B) the presence of *P. eryngii* resulted in microbiota enriched mainly in *Lactobacillus*, *Prevotella*, *Collinsella*, *Bifidobacterium*, *Faecalibacterium* but with a decreased abundance of other genera such as mainly *Dorea*, *Flavonifractor*, *Bilophila* and *Ruminococcus*, (Supplementary Table S3).

**Figure 5:**
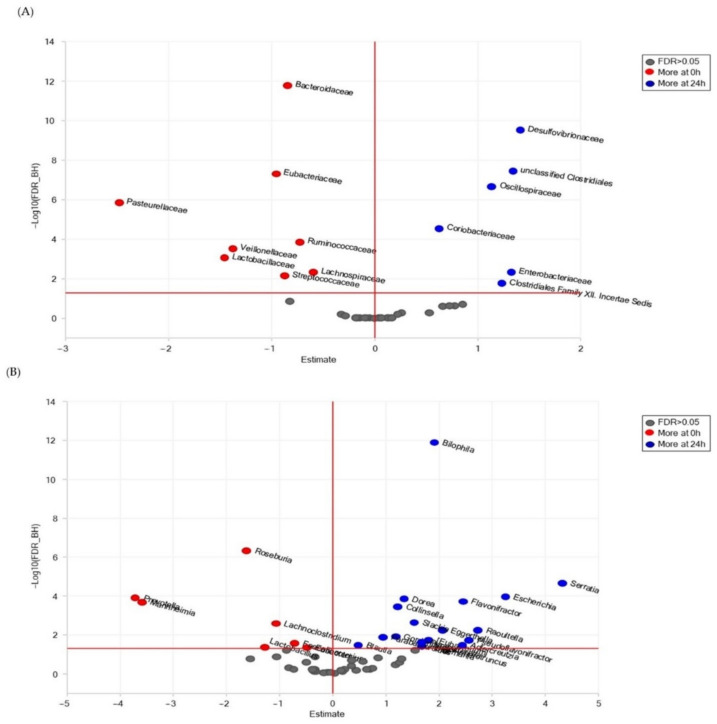
Volcano plots for the comparison of microbiota abundance among fecal samples without the presence of *P. eryngii* and with or without fermentation (NC0 and NC24, respectively). (**A**) families, (**B**) genera. Estimate = log2 fold change; the vertical red line is set to Estimate = 0); the horizontal red line is set to FDR = 0.05.

In order to elucidate microbiota population alterations arising through fermentation only, in the absence of *P. eryngii*, pairwise comparisons were also performed for the NC0 vs. NC24 control groups (Figure 5A and Supplementary Table S5). Four of the most abundant families (*Clostridiales Incertae Sedis* Family XII., *Oscillospiraceae*, unclassified *Clostridiales* and *Desulfovibrionaceae*) and five of the less abundant (*Lactobacillaceae*, *Veillonellaceae*, *Ruminococcaceae*, *Lachnospiraceae* and *Bacteroidaceae*) in NC24 exhibited the same trend when NC24 was compared to PE24 (Figure 4A and Supplementary Table S4)., Analogous trends were observed among genera as indicated through the comparison of Figure 4B vs. Figure 5B and/or Supplementary Table S4 vs. Supplementary Table S5.

### 3.2. Metabolomics Analysis

#### 3.2.1. Overview of the Studied Groups

In the current study, ^1^H NMR spectroscopy was applied for the targeted profiling and absolute quantification of 37 metabolites unambiguously identified in the same studied fecal matrix, as previously described. The metabolites concentration across the sample set was subjected to multivariate statistical analysis. Unsupervised Principal Component Analysis (PCA) illustrated a clear separation of the pre- and post-fermentation samples across the PC1, revealing the metabolic variance arising from the fermentation process (Supplementary Figure S3).

**Figure 6:**
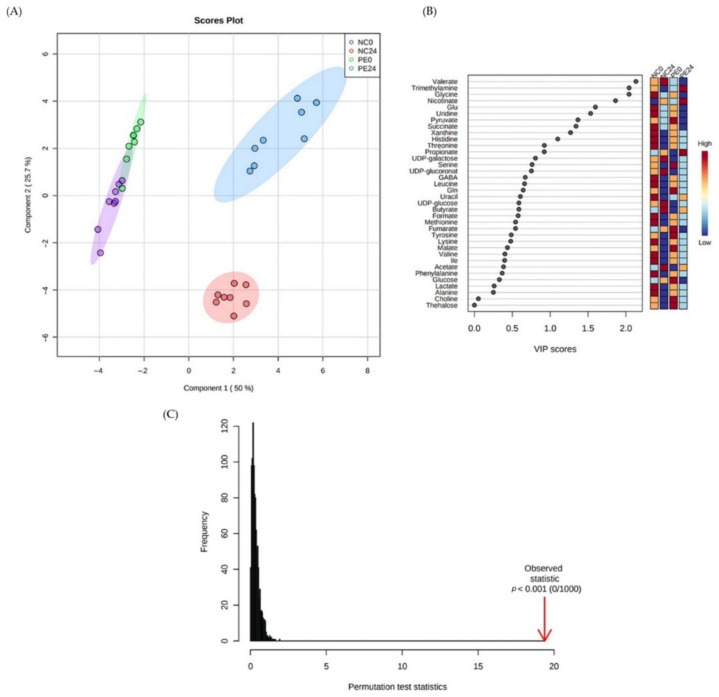
PLS–DA analysis for the pre- and post-fermentation samples from eight volunteers. (**A**) Scores plot of PLS-DA analysis (R2X(cum) = 0.90, Q2(cum) = 0.81, accuracy = 0.97), (**B**) VIPs plot of the studied metabolites. NC0: Samples before fermentation in the absence of an additional carbon source (negative controls at 0 h), NC24: Samples after 24 h of fermentation in the absence of an additional carbon source (negative controls at 24 h), PE0: Samples before fermentation in the presence of lyophilized mushroom powder of *P. eryngii*, PE24: Samples after 24 h of fermentation in the presence of lyophilized mushroom powder of *P. eryngii*, (**C**) Validation of the PLS–DA analysis, by permutation test statistics, indicating that the extracted model is significantly different from a model built on random data. The permutation tests were carried out with 1000 random permutations, thus providing significance of the model at the 0.001 level.

Application of supervised analysis (Partial Least Square Discriminant Analysis, PLS-DA), using the pre/post-fermentation conditions in NC and PE samples as the response variable and the quantified metabolites matrix as the independent variables, confirmed the high degree of separation between the pre- and the post-fermentation conditions across the first component, and further probed the impact of *P. eryngii* fermentation across the second component (Figure 6A) VIPs plot enabled the identification of the most discriminating metabolites (VIP > 1) among the four studied groups. Indicatively, the fermentation in the presence of *P. eryngii* (PE24) is associated with the higher abundance of nicotinate, propionate and TMA while in the absence of the mushroom (NC24) the production of valerate is favored (Figure 6B). The model was validated by permutation test statistics (Figure 6C).

#### 3.2.2. Metabolites Associated with the Addition of *P. eryngii* as Profiled in the Pre-Fermentation Condition

**Figure 7:**
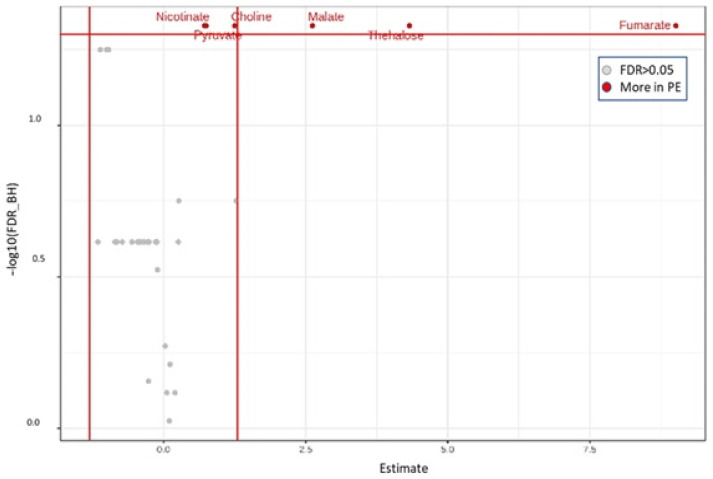
Volcano plots illustrating the changes in metabolite concentrations caused by the *P. eryngii* addition into the pre-fermentation samples (PE0 vs. NC0). Estimate = log2 fold change; the vertical red lines are indicative and are set to fold change 1.5); the horizontal red line is set to FDR = 0.05.

PLS-DA analysis revealed a separation of the pre-fermentation NC0 and PE0 groups along PC2 (Figure 6), which reflects the water-soluble constituents of *P. eryngii* in the PE0 samples. Application of a paired, non-parametric Wilcoxon *t*-test between the two groups revealed six statistically significant metabolites (*p* < 0.05) including glycolysis and Krebs cycle functional metabolites as pyruvate, fumarate, malate, the sugar trehalose, choline and the B complex vitamin nicotinate. All of them are attributed to *P. eryngii* metabolic fingerprinting resulting from the addition of mushroom powder in fecal culture (Figure 7, Supplementary Table S6).

#### 3.2.3. Impact of *P. eryngii* on the Metabolic Profile of the Post-Fermentation Samples

**Figure 8:**
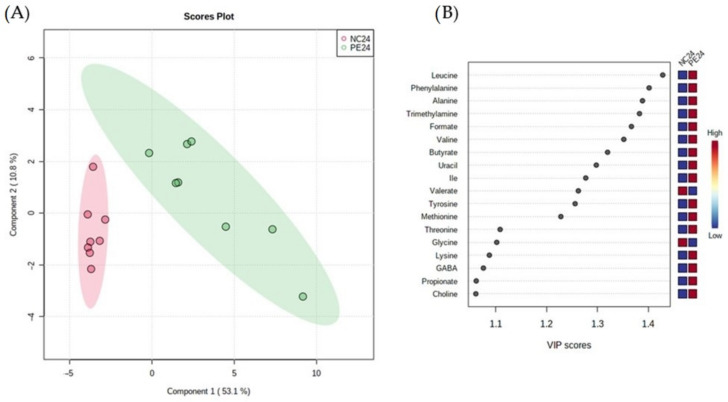
PLS–DA analysis of post- fermentation samples from eight volunteers. (**A**) Scores plot of PLS-DA analysis (R2X(cum) = 0.90, Q2(cum) = 0.69, accuracy = 1.00). (**B**) VIPs plot of the studied metabolites. NC24: Samples after 24 h of fermentation in the absence of an additional carbon source (negative controls at 24 h), PE24: Samples after 24 h of fermentation in the presence of lyophilized mushroom powder of *P. eryngii*.

In order to study the effect of the fermentation of *P. eryngii* by gut microbiota on the fecal metabolome, the metabolite composition of the post-fermentation samples (PE24 vs. NC24) was compared. The PLS-DA model (Figure 8) clearly discriminated the PE samples along the first component when compared to controls (NC24). The analysis highlighted the significantly (VIP > 1) higher levels of certain amino acids as the Branched-Chain Amino Acids (BCAAs) leucine, isoleucine, the aromatic amino acids phenylalanine, and tyrosine, as well as alanine, methionine, threonine, lysine, in the PE samples. The levels of the critical SCFAs butyrate and propionate were also higher, as well as those of the neurotransmitter γ-Aminobutyric acid (GABA), choline and its potential derivatives TMA and formate, and that of the nucleoside uracil. On the other hand, significant (VIP > 1) increased levels of valerate and of the neurotransmitter Gly, were noticed in the control samples (NC24).

**Figure 9:**
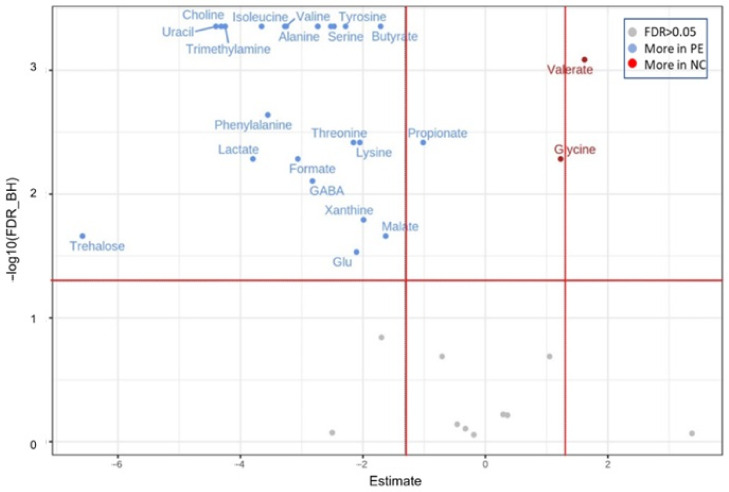
Volcano plots for the pairwise (paired Wilcoxon test) differential metabolic composition of fermented samples with or without the addition of *P. eryngii* (PE24 and NC24 respectively). Estimate = log2 fold change; the vertical red lines are indicative and are set to fold change = 1.5); the horizontal red line is set to FDR = 0.05.

The multivariate PLS-DA results were largely confirmed when univariate non-parametric paired Wilcoxon test was performed (Figure 9, Supplementary Table S7). Amino acids such as leucine, phenylalanine and alanine were more abundant, whereas glycine was less abundant in PE24 samples than their NC24 counterparts. In addition, PE24 samples were richer in SCFAs such as butyrate and propionate, but not in valerate.

## 4. Discussion

This present study focuses on the investigation of *P. eryngii*’s impact on the gut microbiota composition of elderly subjects. The composition of the gut microbiota in the elderly is characterized by reduced bacterial diversity accompanied by a decline in beneficial microorganisms as well as a decrease in the availability of total short-chain fatty acids; the aforementioned are associated with physiological changes in the gastrointestinal tract and a decline in the normal function of the immune system that may contribute to an increased risk of infection and frailty [43,44]. Therefore, the regulation of gut microbiota composition through diet could alleviate frailty, decrease the risk of infection and promote health in the elderly.

The metataxonomic analysis findings, overall, confirm the prebiotic-like properties of *P. eryngii* supporting the outcome of our recent previous studies [34] which, additionally, have established the genoprotective [4] and potent immunomodulating [35] activities of this mushroom. Τhe presence of *P. eryngii* in the in vitro fermentation process led to statistically significant changes in the fecal microbiota composition. Families identified to be significantly more abundant in PE24 post-fermentation samples were *Lactobacillaceae*, *Veillonellaceae*, *Prevotellaceae*, *Acidaminococcaceae*, *Sutterellaceae*, *Bifidobacteriaceae*, *Coriobacteriaceae*, *Lachnospiraceae*, *Ruminococcaceae* and *Bacteroidaceae*. On the contrary, *Clostridiales* Family XII *Incertae* Sedis, *Clostridiales* Family XIII Incertae Sedis, *Oxalobacteraceae*, *Clostridiales* Family XI Incertae Sedis, *Oscillospiraceae*, *Christensenellaceae*, unclassified *Clostridiales*, *Synergistaceae*, *Erysipelotrichaceae*, *Peptostreptococcaceae*, *Streptococcaceae* and *Desulfovibrionaceae* were more abundant in NC24 post-fermentation samples (Figure 4A; Supplementary Table S2). At the genus level, the presence of *P. eryngii* resulted in microbiota enriched in *Lactobacillus*, *Prevotella*, *Anaerostipes*, *Dialister*, *Sutterella*, *Collinsella*, *Bifidobacterium*, *Faecalibacterium*, *Lachnoclostridium* and *Blautia* (Figure 4B; Supplementary Table S3). Most importantly, the genera *Lactobacillus* [45] and *Bifidobacterium* [46], both including widely used probiotic strains, and the *Lactobacillaceae* and *Bifidobacteriaceae* families, respectively, showed an increase in abundance in post-fermentation PE samples, an effect not observed in control NC samples (Figure 4 and Figure 5, Supplementary Tables S2–S5).

Probiotics, i.e., “live microorganisms, which when consumed in adequate amounts, confer a health effect on the host” [10], are already being used to treat or prevent human diseases and have been shown to exert a protective role in the gut; they compete with pathogens to produce direct antimicrobial effects and indirectly enhance intestinal barrier function [47]. A wide range of bacteria belonging primarily to the genera *Bifidobacterium* and *Lactobacillus* could produce bacteriocins or antibacterial proteins highly effective against foodborne pathogens such as *Staphylococcus aureus*, *Pseudomonas fluorescens*, *P. aeruginosa*, *Salmonella typhi*, *Shigella flexneri*, *Listeria monocytogenes*, *Escherichia coli* O157:H7 and *Clostridium botulinum* [48,49], and thus, have a long history of use in food [50]. The probiotic effects of the genus *Lactobacillus*, in particular, have been extensively investigated during the last decade [48]. Clinical studies and animal experiments have demonstrated that there are significant differences in *Lactobacillus* abundance between diseased and disease-free hosts, and that the administration of specific *Lactobacillus* species ameliorates clinical symptoms and/or prevents relapse of several intestinal disorders [48]. Lately, *Lactobacilli* strains have been used in colorectal cancer therapeutics alone or in combination with chemotherapy [51]. In addition, *Lactobacillus* spp. could improve conditions such as gastrointestinal diseases, allergies and liver disease through various mechanisms, such as producing metabolites that can directly inhibit pathogens, exhibiting immunomodulatory effects, and changing the intestinal microbiota [48]. With regard to the genus *Bifidobacterium*, many probiotic *Bifidobacteria* have shown beneficial effects on humans or animals, e.g., anti-infection, anti-depression, regulating the host immune system, and facilitating host nutrition adsorption [46].

*Prevotellaceae*, more abundant in PE24 samples, is a major family in the guts of mammals. *Prevotella* spp. are considered commensal microbes, are often abundant in the digestive tract of people who eat a fiber-rich diet [43] and are able to degrade dietary components such as cellulose and xylan that are not digested by the host, and increase the content of SCFAs in feces, promote food digestion and maximize energy intake [52]. The *Veillonellaceae*, *Lachnospiraceae*, *Ruminococcaceae* families, also more abundant in PE24 samples, are known to contain many butyrate producing species [53]. *Anaerostipes* is a Gram-positive, anaerobic genus which may protect against colon cancer by producing butyric acid and belongs to the family *Lachnospiraceae* (*Eubacteriales*)*,* one of the most abundant taxa in human gut microbiota that ferment diverse plant polysaccharides to SCFAs (butyrate, acetate) and alcohols (ethanol) [54]. As a genus of the *Lachnospiraceae* family, *Blautia* has attracted particular interest recently due to its contribution to alleviating inflammatory diseases and metabolic diseases, for its antibacterial activity against specific microorganisms and its role in biotransformation and crosstalk with other intestinal microorganisms as well as its potential probiotic properties [55]. It is widely distributed in mammalian feces and intestines, and degrades glucose producing mainly acetic acid, succinic acid, lactic acid, and ethanol [55]. It is worthwhile mentioning that despite the reduction in the abundance of *Oscillospiraceae* in PE24 compared to NC24 samples (Figure 2B), at the genus level, the abundance of *Faecalibacterium* and, hence, *Faecalibacterium prausnitzii*, one of the most abundant and important commensal bacteria of the human gut microbiota producing butyrate through the fermentation of dietary fiber [45], is significantly increased (Supplementary Table S3).

Interestingly, in PE post-fermentation samples (PE24) a decrease in bacterial families’ and genera’s abundance was observed, as described by Shannon and Chao1 alpha-diversity indices (Figure 1). This, being in agreement with previous studies [33,56], could be attributed to the targeted promotion of the growth of specific bacterial families/genera induced by the fermentation of the mushroom’s ingredients, leading to their statistically significant increase of abundance in the bulk of fecal microbiota. Hence, the overall evenness of families/genera detected within GM is affected, as shown in Shannon indices, in a more prominent way (Figure 1b,d).

In order to elucidate population alterations in GM arising through fermentation only, in the absence of *P. eryngii*, pairwise comparisons were also performed for the NC0 vs. NC24 control groups. The outcome (Figure 5A and Supplementary Table S4) shows that six families were more abundant in the post-fermentation samples (NC24). Four of the most abundant families (*Clostridiales* Family XII. Incertae Sedis, *Oscillospiraceae*, unclassified *Clostridiales* and *Desulfovibrionaceae*) in NC24 exhibited the same trend when NC24 was compared to PE24. On the other hand, five of the most abundant families (*Lactobacillaceae*, *Veillonellaceae*, *Ruminococcaceae*, *Lachnospiraceae* and *Bacteroidaceae*) in NC0 were further increased significantly in PE24 only. The same trends were also observed among genera for the same comparisons (Figure 5B and Supplementary Table S5). Taking into account that DESeq2 analysis for the families and genera in the pre-fermentation NC0 and PE0 groups showed no difference in microbiota composition between the two conditions (Supplementary Figure S2), the fermentation in the presence of *P. eryngii* seems to selectively enhance families and genera of health-promoting bacteria (among them *Lactobacillaceae* and *Lactobacillus*, respectively), which is not the case when fermentation occurs without an additional carbon source.

The impact of *P. eryngii* on the fecal metabolome was assessed by ΝΜR-based metabolomics. A total of thirty-seven metabolites were identified in the studied substrate, and the combined use of multivariate and univariate analysis revealed the differentiating metabolites across the studied sample sets.

A comparison of the metabolic profiles of the pre-fermentation samples, PE0 vs. NC0, revealed the metabolites that were more abundant in PE0 samples compared to the respective NC0 ones (Figure 7). Trehalose, an abundant disaccharide of edible plants, is known to possess antioxidant properties and protect humans from diseases such as diabetes, liver steatosis, infections, cancer and neurodegenerative diseases [57]. As water-soluble mushroom ingredients, choline and nicotinate are also more abundant in PE0 samples [4]. Nicotinate, also known as niacin (Vitamin B3), and other B-complex vitamins are found in fruit bodies of the *Pleurotus* genus mushrooms [58]. Pyruvate, malate and fumarate are water-soluble constituents of mushrooms involved in the fungal metabolic pathways of glycolysis and the Krebs cycle, thus, their concentration emerges reasonably higher in the PE0 samples [59].

A number of metabolic alterations occurred in post-fermentation samples due to altered fecal microbiota metabolism in the presence of *P. eryngii*. Discrimination analysis coupled with univariate analysis in post-fermentation samples revealed that twenty-three metabolites were upregulated in PE24, while only two metabolites were more abundant in the NC24 samples. In vitro fermentation of *P. eryngii* resulted in a remarkable increase of SCFAs such as butyrate and propionate. Findings in the literature evidence that following their production, SCFAs are rapidly absorbed by intestinal epithelial cells for further use [17]. It is noteworthy that high SCFA levels are associated with improved immune cell function [60,61], type 2 diabetes alleviation [62] and protection against diet-induced obesity [63], while there is evidence supporting their anti-cancer activity [64]. On the contrary, decreased SCFAs production is linked to immunosenescence of the gut in the elderly [65], rendering the preservation or amelioration of the numbers of gut bacteria that produce them essential.

The proteolysis of proteins or peptides not absorbed in the upper part of the digestive system by gut bacteria, results in the production of numerous intermediary metabolites and other end products [20,21,22], mainly SCFAs, ammonia, polyamines, hydrogen sulfide, and phenolic and indolic compounds. These bacterial metabolites can be transported into colonocytes or to other parts of the body via blood circulation and exert various beneficial or deleterious physiological effects on the gut, liver, and other peripheral organs and tissues [66]. *Pleurotus* mushrooms are a significant source of proteins [67], therefore, as expected, microbiome-induced proteolytic metabolites such as BCAAs (leucine, isoleucine, valine, and alanine) were abundant in the post-fermentation PE samples (Figure 8 and Figure 9). In accordance with our findings, a high-protein diet is accompanied by excessive BCAAs production and increased abundance of *Bacteroidaceae* and *Prevotellaceae* [68]. It has to be underscored that increased levels of BCAAs in the serum are considered biomarkers of insulin-resistance but the link on the entrance of intestinal BCAAs in the bloodstream, possibly modulated by the abundance of bacteria lacking the genes for BCAAs’ uptake, is not yet resolved [69]. Besides BCAAs, several amino acids appear to be in abundance in PE24 samples, such as the aromatic tyrosine and phenylalanine, which are precursors of L-DOPA and of the neurotransmitter’s dopamine, norepinephrine and epinephrine. Furthermore, lysine, threonine and methionine concentrations, all of them proposed to derive as end-products through the aspartic acid metabolic degradation, are also elevated in PE24 samples [70]. Interestingly, a recent in silico metabolic profiling prediction of human gut microbiota based on 2856 bacterial genomic data demonstrated that the analyzed genomes of *Bifidobacterium* spp., abundant in PE24 samples, can synthesize all amino acids except cysteine [71], which could be of relevance to the outcome of the present study.

GABA, a neurotransmitter of the central nervous system, is also induced in post-fermentation PE samples. It is well known that both *Lactobacilli* and *Bifidobacteria*, also abundant in post-fermentation PE samples, produce GABA with beneficial effects against the pathogenesis of depression and anxiety through the gut-brain axis [72,73]. It is also reported that GABA can regulate the proliferation of T cells and thus has an immunomodulatory activity [74]. The production of trimethylamine (TMA) was also enhanced in post-fermentation PE samples, very probably due to the anaerobic fecal microbiota metabolism of choline, a water-soluble mushroom ingredient, abundant in PE0 samples. TMA is further absorbed through intestinal epithelium [75] and oxidized in the liver by the flavin-containing monooxygenase (FMO) enzyme family forming trimethylamine N-oxide (TMAO). The latter has been implicated in atherosclerosis and cardiovascular disease due to alterations in cholesterol and bile acid metabolism, and in activation of inflammatory pathways [76]. However, the “Atherosclerosis Risk in Communities” study [77] and the “European Prospective Investigation into Cancer and Nutrition” study [78] did not report increased cardiovascular risk with increasing dietary intake of choline. In addition, fish, which is an important source of trimethylamine in the diet [79], is not associated with risk of cardiovascular disease in the “Physicians’ Health Study” [80]. On the contrary, a meta-analysis concluded that fish consumption is inversely associated with fatal coronary heart disease [81]. Therefore, it is questioned whether TMAO is the mediator or the result of the cardiovascular disease process, and the role of TMAO in human health and diseases is not yet clearly defined [82].

In conclusion, both the metataxonomic and metabolomic analyses confirm the prebiotic-like properties of *P. eryngii*, since its presence in the fermentation process led to statistically significant increase in the abundance of beneficial bacteria (e.g., *Lactobacillaceae* and *Bifidobacteriaceae*) and beneficial metabolites including SCFAs, essential amino acids and neurotransmitters. Our results, overall, support previous studies [34] which, additionally, have established the genoprotective [4] and potent immunomodulating [35] activities of this mushroom and provide substantial evidence supporting the health-promoting effect of this edible mushroom, particularly in the elderly.

## Data Availability

The data presented in the manuscript are available on request from the corresponding authors.

## Supplementary Materials

The following supporting information can be downloaded at: https://www.mdpi.com/article/10.3390/jof9010128/s1, Figure S1. Mean percentage of families as identified by consensus data of all primers and V3 hypervariable region exclusively. Table S1. Families in consensus data of all primers and in V3 hypervariable region exclusively with differential abundance among NC24 and PE24 samples, as identified using DESeq2 paired analysis (Estimate = log2 fold change, FDR < 0.05). Figure S2. Scores plot between the selected PCs resulting from Principal Component Analysis (PCA) of families (A) and genera (B) abundance in NC (negative controls) and PE samples before fermentation. Table S2. Differentially abundant families among NC24 and PE24 samples, as identified using DESeq2 paired analysis (Estimate = log2 fold-change, FDR < 0.05), ranking by the estimate value. Table S3. Differentially abundant genera among NC24 and PE24 samples, as identified using DESeq2 paired analysis (Estimate = log2 fold-change, FDR < 0.05), ranking by the estimate value. Table S4. Differentially abundant families among pre- and post-fermentation negative control samples (NC0 and NC24 respectively) as identified using DESeq2 paired analysis (Estimate = log2 fold-change, FDR < 0.05) ranking by the estimate value. Table S5. Differentially abundant genera among pre and post fermentation negative control samples (NC0 and NC24 respectively) as identified using DESeq2 paired analysis (Estimate = log2 fold-change, FDR < 0.05) ranking by the estimate value. Figure S3. Scores plot of PCA analysis. Illustration of quantified metabolites according to their ^1^H–NMR data of the 8 volunteers. Table S6. Differential metabolites’ concentrations among the pre-fermentation NC0 and PE0 due to the addition of *P. eryngii* lyophilized powder in the fermentation medium, as identified using non-parametric Wilcoxon paired analysis (Estimate = log2 fold-change, FDR < 0.05) ranking by the estimate value. Table S7. Differential metabolites’ concentrations among the post-fermentation NC24 and PE24 as identified using non-parametric wilcoxon paired analysis (Estimate = log2 fold-change, FDR < 0.05) ranking by the estimate value.
