# Supplementary material for: In Vitro Fermentation of Pleurotus eryngii Mushrooms by Human Fecal Microbiota: Metataxonomic Analysis and Metabolomic Profiling of Fermentation Products
Source: J Fungi (Basel). 2023 Jan 16;9(1):128. doi: 10.3390/jof9010128 (PMC9865116; doi:10.3390/jof9010128)
Supplement: Supplementary file 1 [file jof-09-00128-s001.zip › jof-2146254-supplementary.pdf]

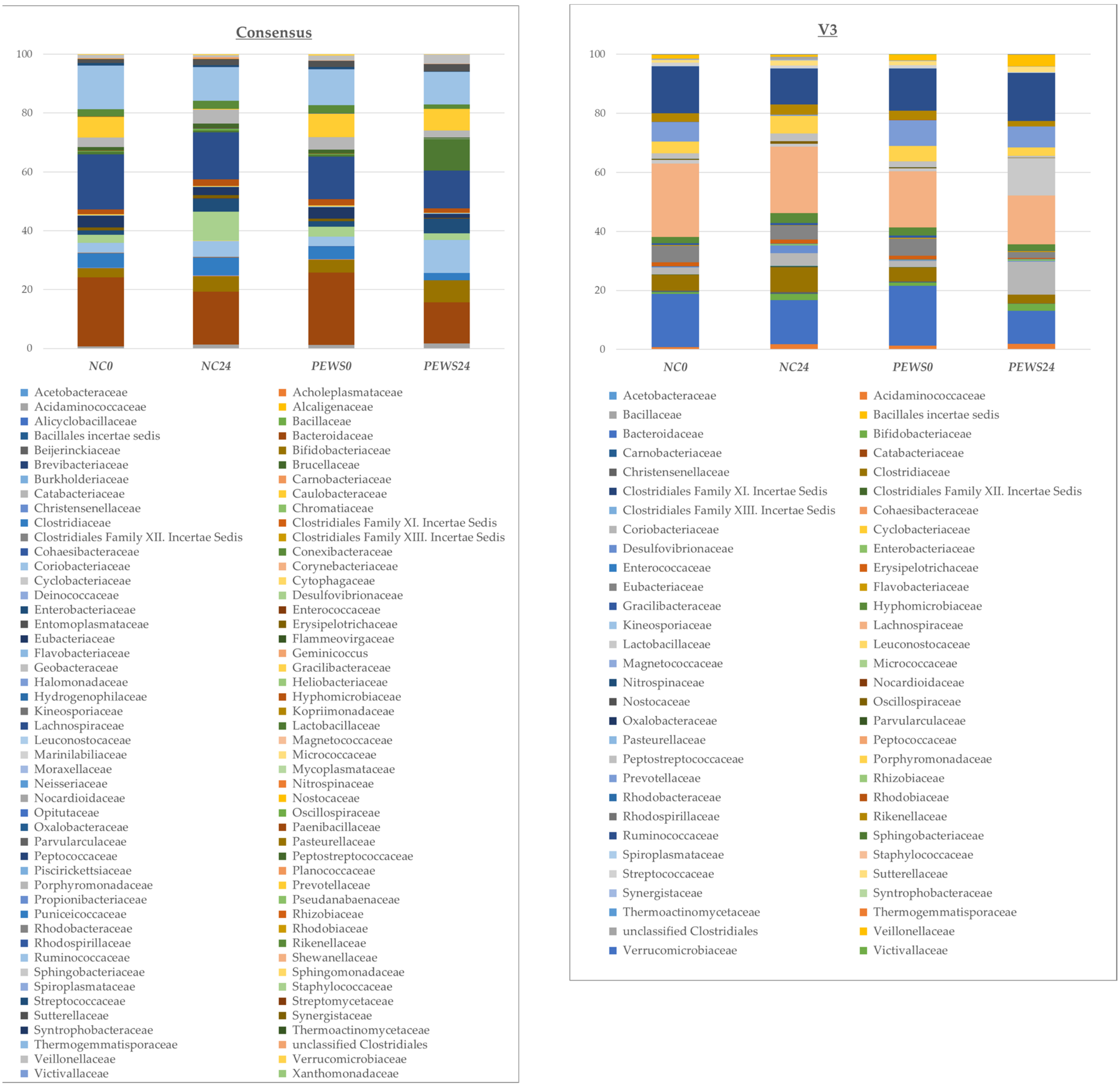

Supplementary Figure S1. Mean percentage of families as identified by consensus data of all primers and V3 hypervariable region exclusively. Consensus detected 96 different families across samples, while V3 primer detected 60 families in the same samples. NC0: Samples before fermentation in the absence of additional carbon source (negative controls at 0 h), NC24: Samples after 24 h of fermentation in the absence of additional carbon source (negative controls at 24 h), PE0: Samples before fermentation in the presence of lyophilized mushroom powder of *P. eryngii*, PE24: Samples after 24 h of fermentation in the presence of lyophilized mushroom powder of *P. eryngii*.

Supplementary Table S1. Families in consensus data of all primers and in V3 hypervariable region exclusively with differential abundance among NC24 and PE24 samples, as identified using DESeq2 paired analysis (Estimate = log2 fold change, FDR<0.05). NC24: Samples after 24 h of fermentation in the absence of additional carbon source, PE24: Samples after 24 h of fermentation in the presence of lyophilized mushroom powder of *P. eryngii.*

	Consensus	V3
**More abundant in NC24**	Christensenellaceae	Christensenellaceae
	Clostridiales Family XI. Incertae Sedis	Clostridiales Family XI. Incertae Sedis
	Clostridiales Family XII. Incertae Sedis	Clostridiales Family XII. Incertae Sedis
	Clostridiales Family XIII. Incertae Sedis	Clostridiales Family XIII. Incertae Sedis
	Desulfovibrionaceae	Desulfovibrionaceae
	Erysipelotrichaceae	Erysipelotrichaceae
	Oscillospiraceae	Oscillospiraceae
	Oxalobacteraceae	Peptococcaceae
	Peptostreptococcaceae	Peptostreptococcaceae
	Streptococcaceae	Streptococcaceae
	Synergistaceae	Synergistaceae
	unclassified Clostridiales	unclassified Clostridiales
**More abundant in PE24**	Acidaminococcaceae	Acidaminococcaceae
	Bacteroidaceae	Bacteroidaceae
	Bifidobacteriaceae	Bifidobacteriaceae
	Coriobacteriaceae	Coriobacteriaceae
	Lachnospiraceae	Lachnospiraceae
	Lactobacillaceae	Lactobacillaceae
	Prevotellaceae	Prevotellaceae
	Ruminococcaceae	Ruminococcaceae
	Sutterellaceae	Sutterellaceae
	Veillonellaceae	Veillonellaceae

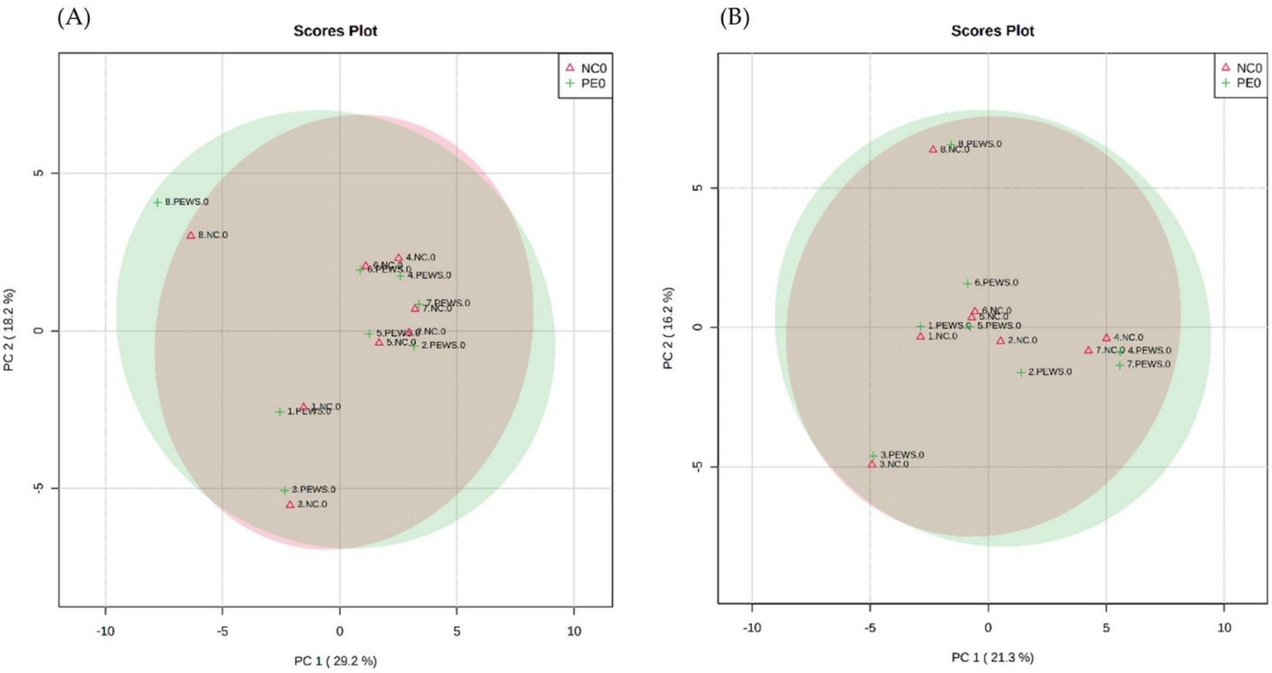

Supplementary Figure S2. Scores plot between the selected PCs resulting from Principal Component Analysis (PCA) of families (A) and genera (B) abundance in NC (negative controls) and PE samples before fermentation. The explained variances are shown in brackets. NC0: Samples before fermentation in the absence of additional carbon source (negative controls at 0 h), PE0: Samples before fermentation in the presence of lyophilized mushroom powder of *P. eryngii*.

Supplementary Table S2. Differentially abundant families among NC24 and PE24 samples, as identified using DESeq2 paired analysis (Estimate = log2 fold-change, FDR<0.05), ranking by the estimate value. NC24: Samples after 24 h of fermentation in the absence of additional carbon source, PE24: Samples after 24 h of fermentation in the presence of lyophilized mushroom powder of *P. eryngii*. NC24 & PE24 Mean (St.Dev): Mean percentage and standard deviation of family's abundance in the groups.

NC24 vs PE24	Family	Estimate	FDR_BH	NC24 Mean (St.Dev)	PE24 Mean (St.Dev)
More abundant in NC	Clostridiales Family XII. Incertae Sedis	-8.391	4.87E-14	0.15% (0.25%)	0.00% (0.00%)
	Clostridiales Family XIII. Incertae Sedis	-4.810	1.72E-05	0.08% (0.07%)	0.00% (0.00%)
	Oxalobacteraceae	-3.827	0.0012	5.37% (4.82%)	0.01% (0.01%)
	Clostridiales Family XI. Incertae Sedis	-3.700	0.0018	0.04% (0.05%)	0.00% (0.00%)
	Oscillospiraceae	-3.173	1.42E-07	0.08% (0.07%)	0.04% (0.03%)
	Christensenellaceae	-1.994	0.002	0.30% (0.30%)	0.06% (0.07%)
	unclassified Clostridiales	-1.772	9.22E-06	6.00% (1.63%)	0.07% (0.06%)
	Synergistaceae	-1.662	0.012	0.30% (0.30%)	0.02% (0.03%)
	Erysipelotrichaceae	-1.513	1.19E-08	0.02% (0.03%)	0.21% (0.32%)
	Peptostreptococcaceae	-1.261	8.61E-05	4.59% (3.39%)	0.32% (0.47%)
	Streptococcaceae	-1.249	0.0017	0.06% (0.10%)	0.12% (0.14%)
	Desulfovibrionaceae	-0.989	0.0142	9.90% (3.51%)	2.16% (0.97%)
More abundant in PE	Lactobacillaceae	6.125	5.55E-13	0.04% (0.05%)	10.75% (13.93%)
	Veillonellaceae	3.500	8.32E-07	0.04% (0.05%)	3.15% (3.22%)
	Prevotellaceae	2.951	0.0486	0.02% (0.03%)	7.40% (15.27%)
	Acidaminococcaceae	2.234	0.0027	1.29% (1.48%)	1.70% (1.41%)
	Sutterellaceae	1.753	0.0009	0.00% (0.00%)	2.45% (1.44%)
	Bifidobacteriaceae	1.660	6.19E-05	5.27% (5.65%)	7.40% (8.41%)
	Coriobacteriaceae	1.403	0.0007	5.37% (4.82%)	11.27% (14.27%)
	Lachnospiraceae	0.808	0.0256	6.00% (1.63%)	12.92% (5.50%)
	Ruminococcaceae	0.805	5.51E-06	5.27% (5.65%)	11.17% (9.73%)
	Bacteroidaceae	0.747	0.0017	18.02% (9.19%)	14.05% (8.31%)

Supplementary Table S3. Differentially abundant genera among NC24 and PE24 samples, as identified using DESeq2 paired analysis (Estimate = log2 fold-change, FDR<0.05), ranking by the estimate value. NC24: Samples after 24 h of fermentation in the absence of additional carbon source, PE24: Samples after 24 h of fermentation in the presence of lyophilized mushroom powder of *P. eryngii*. NC24 & PE24 Mean (St.Dev): Mean percentage and standard deviation of genus' abundance in the groups.

NC24 vs PE24	Genus	Estimate	FDR_BH	NC24 Mean (St.Dev)	PE24 Mean (St.Dev)
More abundant in NC	*Adlercreutzia*	-3.960	0.0285	0.04% (0.09%)	0.00% (0.00%)
	Holdemania	-3.619	0.0118	0.08% (0.08%)	0.01% (0.01%)
	Oscillibacter	-3.485	0.0001	0.38% (0.46%)	0.03% (0.03%)
	Odoribacter	-3.427	0.0163	0.09% (0.09%)	0.02% (0.04%)
	Serratia	-3.270	0.0247	0.04% (0.03%)	0.00% (0.01%)
	[Eubacterium]	-2.993	0.0323	0.13% (0.19%)	0.02% (0.03%)
	Dorea	-2.862	5.35E-06	2.62% (2.97%)	0.23% (0.29%)
	Flavonifractor	-2.232	0.0342	0.35% (0.51%)	0.05% (0.07%)
	Gordonibacter	-2.222	0.0006	0.15% (0.18%)	0.01% (0.02%)
	Streptococcus	-1.632	0.0002	0.83% (0.94%)	0.15% (0.17%)
	Bilophila	-1.520	0.0004	11.98% (4.37%)	2.34% (1.21%)
	Eggerthella	-1.150	0.0473	0.49% (1.17%)	0.14% (0.36%)
	Slackia	-1.066	0.0170	0.34% (0.44%)	0.11% (0.16%)
	[Ruminococcus]	-0.876	0.0228	1.64% (0.99%)	0.51% (0.28%)
	Ruminococcus	-0.812	0.0070	6.06% (2.52%)	2.02% (1.37%)
More abundant in PE	*Lactobacillus*	6.035	4.32E-12	0.81% (1.66%)	13.42% (17.39%)
	Prevotella	4.300	0.0118	0.09% (0.22%)	8.71% (18.21%)
	Anaerostipes	3.206	0.0007	0.01% (0.02%)	0.06% (0.06%)
	Dialister	2.468	0.0001	0.39% (0.50%)	1.01% (1.21%)
	Sutterella	1.990	0.0152	1.44% (1.55%)	2.49% (2.19%)
	Collinsella	1.973	1.69E-06	3.19% (4.14%)	9.38% (12.80%)
	Bifidobacterium	1.433	5.35E-06	6.89% (7.40%)	9.25% (10.52%)
	Faecalibacterium	1.378	4.13E-06	6.10% (2.24%)	11.30% (11.44%)
	Lachnoclostridium	1.305	0.0313	0.35% (0.32%)	0.66% (0.53%)
	Blautia	0.870	0.0070	6.15% (2.04%)	6.68% (4.38%)

Supplementary Table S4. Differentially abundant families among pre- and post-fermentation negative control samples (NC0 and NC24, respectively) as identified using DESeq2 paired analysis (Estimate = log2 fold-change, FDR<0.05) ranking by the estimate value. NC0 & NC24 Mean (St.Dev): Mean percentage and standard deviation of family abundance in the groups.

NC0 vs NC24	Family	Estimate	FDR_BH	NC0 Mean (St.Dev)	NC24 Mean (St.Dev)
More abundant at 0h	Pasteurellaceae	-2.479	1.42E-06	0.12% (0.19%)	0.00% (0.01%)
	Lactobacillaceae	-1.458	0.0009	0.83% (0.79%)	0.04% (0.05%)
	Veillonellaceae	-1.376	0.0003	1.12% (1.10%)	0.04% (0.05%)
	Eubacteriaceae	-0.957	4.86E-08	4.00% (1.45%)	18.02% (9.19%)
	Streptococcaceae	-0.873	0.007	0.94% (1.41%)	0.06% (0.10%)
	Bacteroidaceae	-0.846	1.67E-12	23.50% (11.25%)	18.02% (9.19%)
	Ruminococcaceae	-0.727	0.0001	14.73% (5.24%)	5.27% (5.65%)
	Lachnospiraceae	-0.596	0.0046	18.76% (6.69%)	6.00% (1.63%)
More abundant at 24h	Desulfovibrionaceae	1.412	2.93E-10	2.78% (1.38%)	9.90% (3.51%)
	unclassified Clostridiales	1.341	3.53E-08	0.25% (0.42%)	6.00% (1.63%)
	Enterobacteriaceae	1.325	0.0046	1.41% (1.94%)	4.59% (3.39%)
	Clostridiales Family XII. Incertae Sedis	1.232	0.0169	0.05% (0.09%)	0.15% (0.25%)
	Oscillospiraceae	1.134	2.2E-07	0.26% (0.30%)	0.08% (0.07%)
	Coriobacteriaceae	0.625	2.83E-05	3.36% (4.89%)	5.37% (4.82%)

Supplementary Table S5. Differentially abundant genera among pre and post-fermentation negative control samples (NC0 and NC24 respectively) as identified using DESeq2 paired analysis (Estimate = log2 fold-change, FDR<0.05) ranking by the estimated value. NC0 & NC24 Mean (St.Dev): Mean percentage and standard deviation of genus' abundance in the groups.

NC0 vs NC24	Genus	Estimate	FDR_BH	NC0 Mean (St.Dev)	NC24 Mean (St.Dev)
More abundant at 0h	*Prevotella*	-3.7216	0.0001	7.76% (15.16%)	0.09% (0.22%)
	Mannheimia	-3.5915	0.0002	0.08% (0.17%)	0.01% (0.03%)
	Roseburia	-1.6249	4.66E-07	5.34% (3.75%)	1.87% (1.54%)
	Lactobacillus	-1.2802	0.0432	1.03% (1.01%)	0.81% (1.66%)
	Lachnoclostridium	-1.0674	0.0025	0.69% (0.40%)	0.35% (0.32%)
	Faecalibacterium	-0.7194	0.0269	9.69% (2.80%)	6.10% (2.24%)
	Eubacterium	-0.4896	0.0438	5.14% (2.57%)	3.62% (1.54%)
More abundant at 24h	*Serratia*	4.3167	2.17E-05	0.00% (0.00%)	0.04% (0.03%)
	Escherichia	3.2488	0.0001	0.00% (0.00%)	0.06% (0.10%)
	Raoultella	2.7268	0.0055	0.04% (0.07%)	0.26% (0.31%)
	Pseudoflavonifractor	2.5579	0.019	0.00% (0.00%)	0.02% (0.01%)
	Flavonifractor	2.4532	0.0002	0.07% (0.12%)	0.35% (0.51%)
	Adlercreutzia	2.4362	0.0327	0.00% (0.01%)	0.04% (0.09%)
	Eggerthella	2.0631	0.0056	0.19% (0.49%)	0.49% (1.17%)
	Bilophila	1.9112	1.26E-12	3.31% (1.74%)	11.98% (4.37%)
	[Eubacterium]	1.8029	0.0188	0.07% (0.12%)	0.13% (0.19%)
	Parasutterella	1.673	0.0237	0.38% (0.78%)	1.18% (2.46%)
	Holdemania	1.672	0.0377	0.03% (0.03%)	0.08% (0.08%)
	Slackia	1.5297	0.0023	0.13% (0.21%)	0.34% (0.44%)
	Dorea	1.3411	0.0001	1.12% (1.46%)	2.62% (2.97%)
	Collinsella	1.2181	0.0003	1.76% (3.08%)	3.19% (4.14%)
	Gordonibacter	1.184	0.0119	0.08% (0.12%)	0.15% (0.18%)
	Parabacteroides	0.9427	0.0129	1.79% (1.01%)	3.85% (2.52%)
	Blautia	0.475	0.0327	4.55% (2.02%)	6.15% (2.04%)

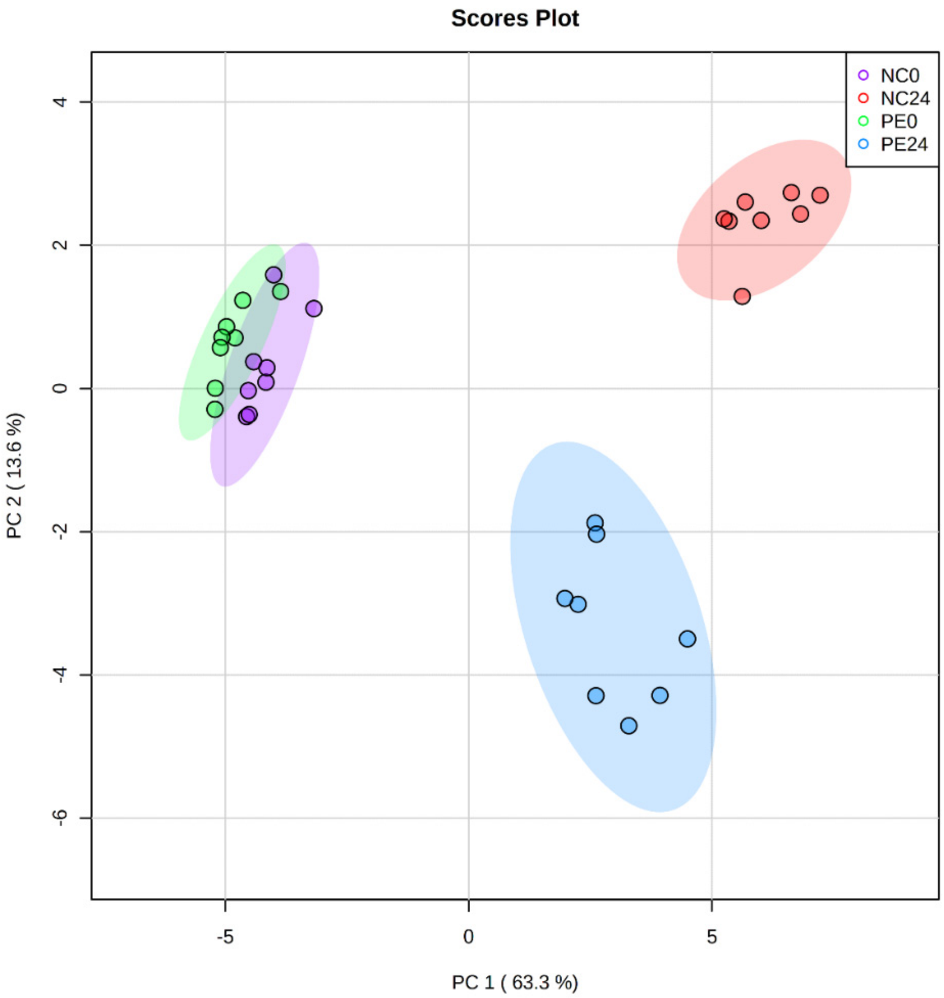

Supplementary Figure S3: Scores plot of PCA analysis. Illustration of quantified metabolites according to their ^{1}H – NMR data of the 8 volunteers. NC0: Samples before fermentation in the absence of additional carbon source (negative controls at 0 h), NC24: Samples after 24 h of fermentation in the absence of additional carbon source (negative controls at 24 h), PE0: Samples before fermentation in the presence of lyophilized mushroom powder of *P. eryngii*, PE24: Samples after 24 h of fermentation in the presence of lyophilized mushroom powder of *P. eryngii*.

Supplementary Table S6. Differential metabolites' concentrations among the pre-fermentation NC0 and PE0 due to the addition of *P.eryngii* lyophilized powder in the fermentation medium, as identified using non-parametric Wilcoxon paired analysis (Estimate = log2 fold-change, FDR<0.05) ranking by the estimate value. NC0: Samples before fermentation in the absence of additional carbon source, PE24: Samples before fermentation in the presence of lyophilized mushroom powder of *P. eryngii*.

Metabolites	Estimate	FDR_BH
Fumarate	9.0141	0.0469
Thehalose	4.3264	0.0469
Malate	2.618	0.0469
Choline	1.2517	0.0469
Pyruvate	0.7467	0.0469
Nicotinate	0.7222	0.0469

Supplementary Table S7. Differential metabolites' concentrations among the post-fermentation NC24 and PE24 as identified using non-parametric wilcoxon paired analysis (Estimate = log2 fold-change, FDR<0.05) ranking by the estimate value. NC24: Samples after 24 h of fermentation in the absence of additional carbon source, PE24: Samples after 24 h of fermentation in the presence of lyophilized mushroom powder of *P. eryngii*.

Direction of comparison	Metabolites	Estimate	FDR_BH
More abundant in PE24 group	Uracil	4.4474	0.0004
	Trimethylamine	4.3001	0.0004
	Choline	3.9622	0.0004
	Phenylalanine	3.6625	0.0004
	Isoleucine	3.6601	0.0004
	Formate	3.3643	0.0004
	Alanine	3.312	0.0004
	Valine	3.258	0.0004
	Lactate	2.8956	0.0004
	Leucine	2.7731	0.0004
	GABA	2.746	0.0004
	Methionine	2.6026	0.0004
	Lysine	2.5106	0.0023
	Nicotinate	2.4606	0.0038
	Serine	2.2928	0.0038
	Tyrosine	2.2403	0.0038
	Threonine	2.0256	0.0052
	Glutamine	1.9606	0.0052
	Butyrate	1.7769	0.0078
	Malate	1.563	0.0160
	Xanthine	1.3622	0.0219
	Propionate	0.9691	0.0219
More abundant in NC24 group	Glycine	-1.4756	0.0052
	Valerate	-1.69	0.0008
